# Lethal Encephalitis in Seals with Japanese Encephalitis Virus Infection, China, 2017

**DOI:** 10.3201/eid2508.181663

**Published:** 2019-08

**Authors:** Xiangdong Li, Mingming Qiao, Xiaoyu Deng, Xi Chen, Shengyong Sun, Qian Zhang, Wenjie Zhang, Feifei Tan, Zhe Sun, Xizhao Chen, Ming Sun, Kegong Tian

**Affiliations:** National Research Center for Veterinary Medicine, Luoyang, China (X. Li, F. Tan, Z. Sun, K. Tian);; Beijing Anheal Laboratories Co. Ltd, Beijing, China (M. Qiao, X. Deng, Xi Chen, S. Sun, Q. Zhang, W. Zhang, Xizhao Chen, M. Sun);; Henan Agricultural University, Zhengzhou, China (K. Tian)

**Keywords:** Japanese encephalitis virus, seal, encephalitis, China, vector-borne infections, viruses, meningitis/encephalitis, immunohistochemistry, electron microscopy, genotype I, zoonoses

## Abstract

We isolated Japanese encephalitis virus (JEV) from brain samples of 2 seals with lethal encephalitis at Weihai Aquarium, Weihai, China, in 2017. We confirmed our findings by immunohistochemical staining and electron microscopy. Phylogenetic analysis showed this virus was genotype I. Our findings suggest that JEV might disseminate though infected zoo animals.

Japanese encephalitis is a mosquitoborne zoonotic viral disease caused by Japanese encephalitis virus (JEV; family *Flaviviridae*, genus *Flavivirus*). The virus genome contains a 5′ untranslated region (UTR), followed by a 10,296-nt coding region and a 3′ UTR. The polyprotein consists of 3 structural proteins designated capsid, membrane, and envelope (E) and 7 nonstructural proteins (NS1, NS2A, NS2B, NS3, NS4A, NS4B, and NS5) (*1*). JEVs are classified into 5 genotypes (I–V) on the basis of the E gene sequence. Of these 5 genotypes, genotype I is the main type circulating in Asia (*2*).

JEV is transmitted primarily through *Culex* spp. mosquitoes and infects birds and some livestock species (*3*). Humans can also become infected but are dead-end hosts for JEV. Viral encephalitis can develop after JEV infection in humans and has a 30% mortality rate. Survivors have permanent neurologic sequelae, making JEV a clinically significant pathogen for viral encephalitis in Asia (*4*). Pigs are the reservoir hosts of JEV and play a role in transmitting this virus to humans. Pigs infected with JEV show pyrexia and anorexia, and pregnant pigs infected with JEV have an increased risk for stillborn offspring and offspring with congenital deformities (*5*). Horses and cattle can also become infected with JEV and occasionally show neurologic signs (*6*,*7*). Besides humans and the previously mentioned animals, JEV has rarely been reported in other mammals. In this study, we investigate the causative agent of lethal viral encephalitis in 2 aquatic mammalian seals.

## The Study

**Figure 1:**
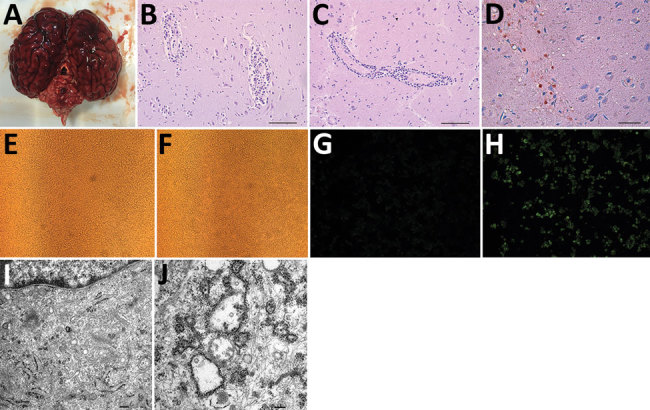
Pathologic examination and virus culturing of brain tissue samples from 2 seals with lethal encephalitis infected with Japanese encephalitis virus (JEV), China, 2017. A) Hemorrhaging seal brain. B) Histochemical staining of a glial nodule in the cerebrum showing lymphocyte infiltration around small blood vessels. Scale bar represents 50 μm. C) Histochemical staining of tissue showing coalescing nonsuppurative encephalitis with neuronal degeneration and perivascular cuffing. Scale bar represents 50 μm. D) Immunohistochemical staining of tissue with anti-JEV polyclonal mouse serum showing JEV antigen. Scale bar represents 20 μm. E–H) Baby hamster kidney cell line BHK-21 incubated with (F, H) and without (E, G) JEV Seal-Anheal-2017 (i.e., brain homogenate supernatant from infected seals passaged through BHK-21 cells 4 times) fluorescently stained by using anti-JEV polyclonal mouse serum (G, H). Cells incubated with the fourth passage of brain homogenate demonstrated cytopathic effect (F) and fluorescence (H), indicating the presence of JEV antigen. Original magnification ×200. I, J) Electron microscopic images of BHK-21 cells not infected (I) and infected (J) with Seal-Anheal-2017. Many mature virions and proliferative vesicles were observed in the endoplasmic reticulum of JEV-infected cells (J). Scale bars represent 0.2 μm.

In 2017, 2 speckled seals (*Phoca hispida*), a 1-year-old male seal and 1.5-year-old female seal, kept in Weihai Aquarium (Weihai, Shandong Province, China) were found displaying neurologic signs (backward head). The rest of the seals (3 male, 9 female, 2–8 years of age) in this aquarium did not display any clinical signs of illness. All seals were raised in the same pools and fed a diet of frozen smashed fish. The 2 sick seals were found dead the next day; immediately afterward, we performed necropsies. We observed hemorrhages in the brain (Figure 1, panel A) but found no lesions in other organs or tissues.

We collected tissue samples to test for seal parvovirus, phocine distemper virus, influenza A virus, coronavirus, canine distemper virus, canine adenovirus, pseudorabies virus, rabies virus, and JEV using commercial real-time PCR kits (Beijing Anheal Laboratories Co. Ltd., http://www.anheal.com). PCR results were negative for all tested pathogens except JEV, indicating this virus might have infected these seals.

We collected brain samples and fixed them in 10% buffered formalin for histopathologic analysis. We embedded samples in paraffin, sectioned them, and stained them with hematoxylin and eosin according to standard protocols. We used anti-JEV polyclonal mouse serum (kindly provided by the Department of Viral Encephalitis, National Institute for Viral Disease Control and Prevention, Beijing, China) to detect viral antigen in brain samples and performed immunohistochemical staining as previously described (*8*). Histopathologic analysis of seal brain samples showed lymphocyte infiltration around small blood vessels and typical nonsuppurative encephalitis (Figure 1, panels B, C). Immunohistochemical staining with anti-JEV polyclonal mouse serum indicated JEV antigen in seal brain samples (Figure 1, panel D), confirming infection with JEV.

We passaged supernatants of brain homogenates from infected seals on baby hamster kidney cell line BHK-21. We cultured cells in Dulbecco-modified Eagle medium supplemented with 10% (vol/vol) calf serum, 100 U/mL penicillin, and 100 mg/mL streptomycin at 37°C in 5% CO_2_. We observed cytopathic effects (cell swelling and detachment, intercellular space dilatation) with the fourth passage of brain homogenate supernatant (Figure 1, panels E, F). Immunofluorescent staining of these cells with anti-JEV polyclonal mouse serum revealed JEV antigen (Figure 1, panels G, H).

We subjected this fourth passage of virus to negative-staining electron microscopy (EM) analysis. We ultracentrifuged cell supernatant at 82,000 × *g* for 30 min, pelleted virus onto 3-mm grids, and then stained grids with 2% sodium phosphotungstate for 1.5 min. We used the Tecnai G2 BioTWIN Transmission Electron Microscope (FEI Company, https://www.fei.com) operating at 85 kV to visualize virus particles. We observed round-shaped virions morphologically similar to those of the *Flavivirus* genus (data not shown). Compared with uninfected cells, many mature virions were found in the endoplasmic reticulum of infected cells by transmission EM (Figure 1, panels I, J). We also observed the ultrastructural pathologic change of proliferative vesicles in the endoplasmic reticulum, which were likely induced by JEV infection.

We extracted total RNA from the supernatant of this fourth passage of virus, which we designated Seal-Anheal-2017, using the NucleoSpin RNA Midi kit (TaKaRa Bio Inc., http://www.takara-bio.com), reverse transcribed RNA to cDNA, and amplified the cDNA by PCR using 15 different primer pairs (Table). We obtained the 5′ and 3′ UTR sequences using the 5′ RACE and 3′ RACE Systems for Rapid Amplification of cDNA Ends kits (Invitrogen, https://www.thermofisher.com). Sequence analysis indicated that the complete genome sequence of JEV Seal-Anheal-2017 (GenBank accession no. MH165313) was 10,965 nt in length, encoding a 10,299-nt single open reading frame flanked by a 96-nt 5′ UTR and 570-nt 3′ UTR.

**Table:** Primers used to amplify the full-length genome of JEV isolate Seal-Anheal-2017 obtained from a seal with lethal encephalitis, China, 2017*

Primer pair no.	Primer name	Primer binding site	Length of fragment amplified, bp
1	JEV-1F	1–26	879
	JEV-1R	878–855	
2	JEV-2F	805–823	833
	JEV-2R	1,637–1,615	
3	JEV-3F	1,559–1,580	703
	JEV-3R	2,261–2,242	
4	JEV-4F	2,151–2,170	827
	JEV-4R	2,977–2,959	
5	JEV-5F	2,898–2,917	779
	JEV-5R	3,676–3,658	
6	JEV-6F	3,582–3,599	740
	JEV-6R	4,321–4,300	
7	JEV-7F	4,204–4,224	919
	JEV-7R	5,122–5,104	
8	JEV-8F	5,005–5,024	934
	JEV-8R	5,938–5,918	
9	JEV-9F	5,841–5,858	939
	JEV-9R	6,779–6,757	
10	JEV-10F	6,676–6,697	880
	JEV-10R	7,555–7,537	
11	JEV-11F	7,431–7,451	817
	JEV-11R	8,247–8,228	
12	JEV-12F	8,121–8,139	875
	JEV-12R	8,995–8,974	
13	JEV-13F	8,924–8,946	866
	JEV-13R	9,789–9,771	
14	JEV-14F	9,653–9,675	827
	JEV-14R	10,479–10,460	
15	JEV-15F	10,217–10,236	769

*JEV, Japanese encephalitis virus.

**Figure 2:**
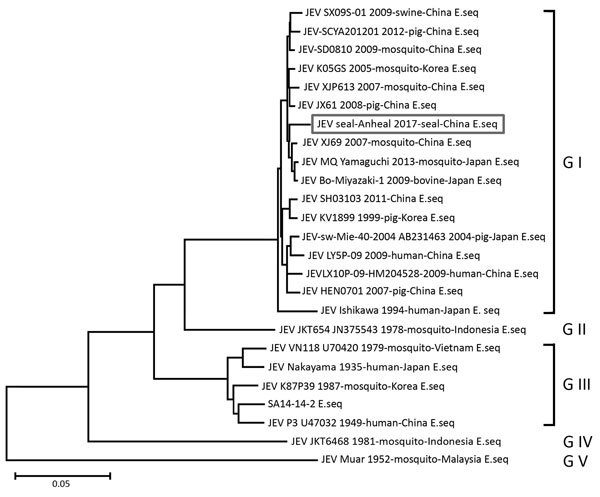
Phylogenetic analysis of JEV E gene of Seal-Anheal-2017 isolate from a seal with lethal encephalitis, China, 2017, compared with 24 JEV strains of different genotypes and species origins. We constructed the tree using the neighbor-joining method and MEGA5.05 (https://www.megasoftware.net). Reliability of the branching orders was evaluated by the bootstrap test (n = 1,000). E, envelope; G, genotype; JEV, Japanese encephalitis virus; seq, sequence. Scale bar indicates nucleotide substitutions per site.

We performed a phylogenetic analysis of the E gene sequence of Seal-Anheal-2017 with those of 24 JEV strains of different genotypes. Results showed that Seal-Anheal-2017 grouped with genotype I, sharing the highest nucleotide sequence homology (99.09%) with XJ69, a mosquito-origin JEV isolated in Xianju County of Zhengjiang Province, China (Figure 2) (*9*). XJ69 and Seal-Anheal-2017 differ by 11 aa: R77Q in membrane; R111G in E; D175N in NS1; Y127S in NS2A; S14L, K26M, and L586M in NS3; I2V in NS4A; K20R in NS4B; and S429G and I721V in NS5. In contrast, SA14-14-2, an attenuated JEV vaccine strain used in humans and pigs in China, differs from Seal-Anheal-2017 at 79 aa (data not shown).

The JEV E protein is a major structural protein contributing to viral virulence, host tropism, and antigenicity (*10*,*11*); in this protein, there are 13 aa substitutions between Seal-Anheal-2017 and SA14-14-2 and 1 between Seal-Anheal-2017 and XJ69. The 8 key amino acid residues that determine virulence did not differ between Seal-Anheal-2017 and XJ69 (data not shown), indicating that Seal-Anheal-2017 might possess typical characteristics of virulent JEV.

## Conclusions

Our data indicate that the causative agent of these 2 seal deaths was JEV. Why and how disease developed is unclear. In humans, young children are susceptible to JEV infection and often have fatal outcomes. Because the 2 seals that died were in their adolescence and the others with no clinical signs were older, age might have contributed to the fatal outcomes seen with these seals.

*Culex tritaeniorhynchus* mosquitoes were found in the aquarium, and seal keepers reported that they had often been bitten by mosquitoes. No human cases of Japanese encephalitis were reported in the vicinity of the aquarium; however, most of the population received compulsory JEV vaccination. *C. tritaeniorhynchus* mosquitoes also feed on livestock and birds, which act as bridging vectors leading to infections in other animals (*12*). A number of pig farms are located several miles away from the aquarium, but wild pigs had never been reported in the area of the aquarium. Therefore, the virus might have been transmitted through the bite of mosquitoes infected with JEV. In summary, we confirmed JEV genotype I as the causative agent of 2 cases of lethal viral encephalitis in seals, on the basis of results from pathologic examinations, virus isolation, immunostaining, and EM.

JEV might cause neurologic disease in more organisms than previously thought. Zoo staff should determine the causative agents in animals displaying neurologic disease, so they can better protect their animals from pathogens. Seals might need to be screened for JEV before they are transported to non–JEV-endemic areas.

## References

[1] Mackenzie JS, Gubler DJ, Petersen LR. Emerging flaviviruses: the spread and resurgence of Japanese encephalitis, West Nile and dengue viruses. Nat Med. 2004;10(Suppl):S98–109. 10.1038/nm1144 15577938

[2] Pan XL, Liu H, Wang HY, Fu SH, Liu HZ, Zhang HL, et al. Emergence of genotype I of Japanese encephalitis virus as the dominant genotype in Asia. J Virol. 2011;85:9847–53. 10.1128/JVI.00825-11 21697481 PMC3196406

[3] Weaver SC, Reisen WK. Present and future arboviral threats. Antiviral Res. 2010;85:328–45. 10.1016/j.antiviral.2009.10.008 19857523 PMC2815176

[4] Zhang JS, Zhao QM, Guo XF, Zuo SQ, Cheng JX, Jia N, et al. Isolation and genetic characteristics of human genotype 1 Japanese encephalitis virus, China, 2009. PLoS One. 2011;6:e16418. 10.1371/journal.pone.0016418 21283590 PMC3026811

[5] Yuan L, Wu R, Liu H, Wen X, Huang X, Wen Y, et al. Tissue tropism and molecular characterization of a Japanese encephalitis virus strain isolated from pigs in southwest China. Virus Res. 2016;215:55–64. 10.1016/j.virusres.2016.02.001 26851509

[6] Katayama T, Saito S, Horiuchi S, Maruta T, Kato T, Yanase T, et al. Nonsuppurative encephalomyelitis in a calf in Japan and isolation of Japanese encephalitis virus genotype 1 from the affected calf. J Clin Microbiol. 2013;51:3448–53. 10.1128/JCM.00737-13 23885004 PMC3811627

[7] Mansfield KL, Hernández-Triana LM, Banyard AC, Fooks AR, Johnson N. Japanese encephalitis virus infection, diagnosis and control in domestic animals. Vet Microbiol. 2017;201:85–92. 10.1016/j.vetmic.2017.01.014 28284628

[8] Tian K, Yu X, Zhao T, Feng Y, Cao Z, Wang C, et al. Emergence of fatal PRRSV variants: unparalleled outbreaks of atypical PRRS in China and molecular dissection of the unique hallmark. PLoS One. 2007;2:e526. 10.1371/journal.pone.0000526 17565379 PMC1885284

[9] Xie RH, Fu SH, Cheng YK, Xu F, Yao PP, Weng JQ, et al. [Isolation and identification of Japanese encephalitis virus from mosquitoes in Zhejiang province]. Zhonghua Liu Xing Bing Xue Za Zhi. 2008;29:712–5. 19031767

[10] Nam JH, Chung YJ, Ban SJ, Kim EJ, Park YK, Cho HW. Envelope gene sequence variation among Japanese encephalitis viruses isolated in Korea. Acta Virol. 1996;40:303–9. 9171460

[11] Fan JM, Luo J, Zhang GP, Chen L, Teng M, Yang MF, et al. Identification and characterization of Japanese encephalitis virus envelope protein gene from swine. Lett Appl Microbiol. 2010;51:11–7. 10.1111/j.1472-765X.2010.02850.x 20477964

[12] Pearce JC, Learoyd TP, Langendorf BJ, Logan JG. Japanese encephalitis: the vectors, ecology and potential for expansion. J Travel Med. 2018;25(suppl_1):S16–26. 10.1093/jtm/tay009 29718435

